# Dental Diseases Increase Risk of Aortic Arch Calcification Independent of Renal Dysfunction in Older Adults: Shenzhen Community Cohort Study

**DOI:** 10.3390/metabo12121258

**Published:** 2022-12-14

**Authors:** Li Yin, Zhengzhipeng Zhang, Changming Xie, Dongling Luo, Wanbing He, Suli Huang, Hui Huang

**Affiliations:** 1 Department of Cardiology, The Eighth Affiliated Hospital, Sun Yat-sen University, Shenzhen 518033, China; 2 Joint Laboratory of Guangdong-Hong Kong-Macao Universities for Nutritional Metabolism and Precise Prevention and Control of Major Chronic Diseases, Sun Yat-sen University, Shenzhen 518038, China; 3 Department of Cardiology, Sun Yat-sen Memorial Hospital, Sun Yat-sen University, Guangzhou 510120, China; 4 Shenzhen Center for Disease Control and Prevention, Shenzhen 518055, China

**Keywords:** aortic arch calcification, dental diseases, renal dysfunction

## Abstract

Many studies have documented that dental diseases were associated with an increased risk of cardiovascular diseases. Aortic arch calcification (AoAC) is a powerful predictor of cardiovascular diseases. However, whether the status of dental health is associated with AoAC is still unknown. 9463 participants over the age of 60 from Shenzhen community centers were included in the cross-sectional analysis. Physical examination data, blood biochemical tests, and AoAC scores calculated by chest radiography were collected and analyzed. Among them, 2630 participants were followed up for AoAC progression up to 36 months. Participants with AoAC suffered more tooth loss than those without AoAC (77.62% vs. 72.91%; *p* < 0.001). Association rule analysis suggested a strong association between dental diseases and AoAC. Tooth loss or decay increased the risk of AoAC progression (HR 1.459; 95%CI 1.284–1.658) after adjusting other risk factors including renal dysfunction. Dental diseases are potential predictors for AoAC in elderly people, which are independent of renal dysfunction.

## 1. Introduction

Cardiovascular disease (CVD) is the leading cause of mortality worldwide. The WHO estimated that, by 2030, CVD would account for more than 23 million deaths per year [1,2]. Aortic artery calcification (AoAC) is recognized as an independent and strong predictor of CVD and major adverse cardiovascular events (MACE) [3]. Epidemiological studies have shown that AoAC increases the risk of cardiovascular events within 10 years by more than three times [4]. AoAC impairs the compliance of the aorta severely and leads to hemodynamic disorder, which further increases left ventricular afterload, decreases the perfusion of coronary artery and organs with high blood flow such as the brain and kidney [5]. The prevalence of AoAC grows with age [6]. Unfortunately, there is still no promising treatment for reversing AoAC so far. Therefore, it is important to explore the potential risk factors and mechanisms of AoAC for early prevention and treatment.

Dental diseases, especially tooth loss and tooth decay, have been shown to be strongly associated with CVD through a variety of mechanisms, including impairing renal function, metabolic homeostasis, and promoting inflammation. Observational studies have found that 90% of renal disease patients suffer from oral symptoms such as tooth loss [7]. Other research documents that tooth loss showed a strong and dose-dependent association with end-stage renal disease [8,9]. The severity of tooth loss may be considered an independent risk indicator for renal dysfunction (eGFR < 60 mL/min/1.73 m^2^) [8]. The prevalence of vascular calcification in patients with CKD stages 3–5 has been shown to be as high as 79% [10]. However, it remains unclear whether dental diseases could lead to AoAC.

In this study, we conducted the investigation of the association between dental diseases and AoAC based on a community-based population and explored possible mechanisms.

## 2. Materials and Methods

### 2.1. Study Population and Data Collection

This population-based study enrolled 10,385 community residents aged over 60 years from Shenzhen Futian district, China, between 2016 and 2019. Demographic, lifestyles, medical history, and physical examination were collected by the electronic medical records. A number of 729 participants were excluded for incomplete chest radiography (X-ray) images for the assessment for AoAC. Eventually, 9463 participants were included in the cross-sectional analysis. Among them, 2630 participants with more than two X-ray aortic calcification scores results and blood biochemical tests were included in the longitudinal analysis. Other excluding criteria include trauma in the jaws and orthodontic treatment, implants and wisdom teeth, history of rheumatologic diseases, or malignancy including adrenocortical carcinoma. All subjects or their agents provided written informed consent. The methods complied with the ethical principles of the Declaration of Helsinki. This study was reviewed and approved by the Ethics Committees of the Eighth Affiliated Hospital, Sun Yat-sen University.

### 2.2. Demographic Characteristics and Baseline Diameters Measurement

Information was collected with structured standardized community health examination forms and face-to-face interviews by trained interviewers. All participants received a physical examination. Blood samples were collected by peripheral venipuncture for all participants and tested for hematology and serum biochemistry. The arterial blood pressure is measured by a doctor using a mercury sphygmomanometer according to a standardized blood pressure measurement plan, and the same arm is used to measure the blood pressure for each patient 3 times in a row [11]. The estimated glomerular filtration rate (eGFR) was calculated from serum creatinine levels with the adjusted CKD-EPI equation for Chinese [12]. Body mass index (BMI) is calculated as weight divided by the square of height (kg/m^2^). Current smoking and drinking were self-reported by participants. History of hypertension, diabetes, and hyperlipidemia was from previous diagnosis by physician and based on biochemical results.

### 2.3. Radiographic Evaluation

AoAC score was measured as calcification in the aortic node region on X-ray. Based on these X-rays, two radiologists who were blind to the personal information of each individual diagnosed the presence of AoAC and calculated the scores, the intraclass correlation coefficient was 0.79, which indicated well consistency [13]. The aortic arch area was divided into 16 equally divided sectors, each sector containing calcified plaque were counted and divided by 16, and the results were expressed as a percentage to indicate the severity of aortic calcification. The detailed method was reported previously [14]. Inconsistent results were calculated by the average of two radiologists as the final result. For participants with multiple records, the AoAC progression was defined as a higher AoAC score than at the baseline.

### 2.4. Dental Examination

The dental examination included an examination of tooth loss and tooth decay, and collection of history of dental diseases including gingivitis and periodontitis. Tooth loss and tooth decay were visually inspected and recorded by experienced dentists, which excludes implants and wisdom teeth [15].

### 2.5. Statistical Analysis

Baseline characteristics of participants were described with the mean ± standard deviation (SD) for normally distributed continuous variables, the median (25th, 75th percentile) for non-normally distributed continuous variables, and percentages for categorical variables. Student-t-test, Mann–Whitney U test, Kruskal–Wallis test, and chi-square test were used for comparison between groups.

The Apriori algorithm of association rules was applied to examine the association between potential risk factors and AoAC. Univariate and multivariate logistic analyses were used to investigate the association between tooth loss, tooth decay, and AoAC. The Cox proportional hazards regression was used to explore whether tooth loss or decay affects the hazard of AoAC progression. IBM SPSS Statistics v26 and R were used for data analyses. For all statistical tests, two-tail *p*-value < 0.05 was the threshold of statistical significance.

## 3. Results

### 3.1. Clinical Characteristics of Participants with or without AoAC

The data-screening process is shown in Figure S1. Participants who did not receive X-ray (*n* = 729), with missing or incorrect records (*n* = 193) were excluded. Finally, 9463 participants were included in the cross-sectional analysis. The cross-sectional analysis was based on the earliest visit of each individual, and 2630 participants with multiple records were analyzed for the AoAC progression over time.

The clinical characteristics of 9463 participants are shown in Table 1. 8031 were diagnosed with AoAC by X-ray. The mean age of participants was 70.76 years, and 57.5% of them were women. Participants with AoAC tended to be older, female. Interestingly, participants with AoAC were found to be more likely to have tooth loss in dental inspections. In the AoAC group, the proportion of tooth loss is 4.71% higher than non-AoAC, and 1.03% for tooth decay.

Additionally, we found higher systolic blood pressure and fasting plasma glucose in the AoAC group, and participants in the AoAC group were more likely to be diagnosed with hypertension and hyperlipidemia compared to those without AoAC.

### 3.2. Association Rule Analysis Suggests a Strong Association between Dental Diseases and AoAC

**Figure 1:**
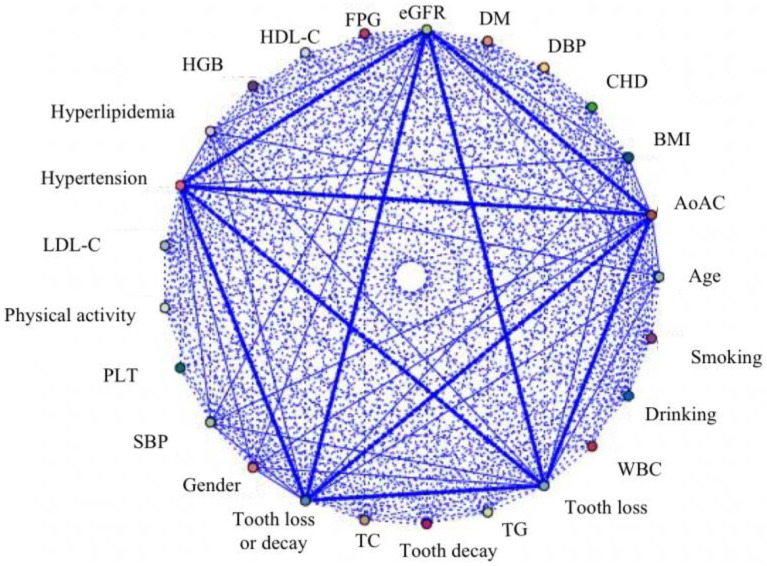
Visualization of the association rules based on the Apriori algorithm. The thickness of lines indicates the strength of the association rules. SBP indicates systolic blood pressure; DBP: diastolic blood pressure; BMI: body mass index; CHD: coronary heart disease; WBC: white blood cell count; HGB: hemoglobin; PLT: platelet; TC: total cholesterol; TG: triglyceride; LDL-C: low-density lipoprotein cholesterol; HDL-C: high-density lipoprotein cholesterol; FPG: fasting plasma glucose; eGFR: estimated glomerular filtration rate.

To further explore potential risk factors associated with AoAC, correlations between 25 clinical indexes were analyzed. Tooth loss, tooth decay, and tooth loss or decay are strongly associated with AoAC (Figure 1). A total of 12 effective association rules were screened as shown in Supplementary Table S1. We identified effective association rules between dental diseases and AoAC, which are stronger than traditional risk factors such as renal dysfunction, smoking, and diabetes.

### 3.3. Dental Diseases Were Independent Risk Factors for AoAC

**Figure 2:**
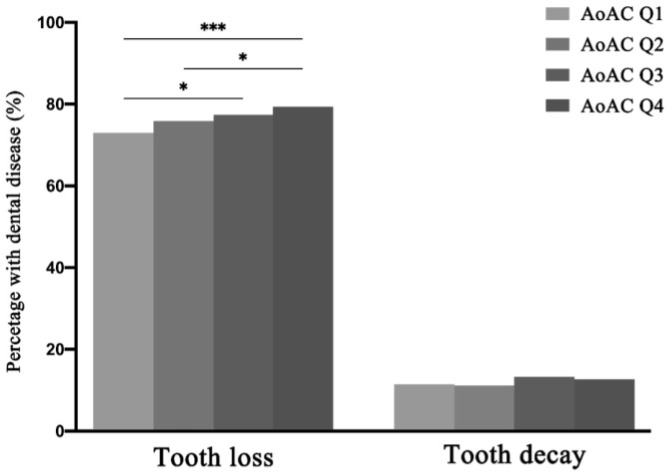
The proportion of participants with tooth loss or tooth decay in each group divided with quartiles of AoAC scores. * Bonferroni-adjusted *p* < 0.05, *** Bonferroni-adjusted *p* < 0.001.

To further explore the relation between tooth loss and tooth decay with AoAC. Participants were divided into four groups according to the quartiles of the AoAC score to explore the distribution of tooth loss and tooth decay between groups. The severity of AoAC gradually increased from Q1 to Q4. The proportion of participants with tooth loss or tooth decay in each group is shown in Figure 2. The proportion of participants with tooth loss (Q1, 72.91%; Q2, 75.90%; Q3, 77.36%; Q4, 79.33%) was significantly different among four groups (*χ*^2^ = 23.56, *p* < 0.001). The results of the Mantel–Haenszel test (*χ*^2^ = 23.56, *p* < 0.001) showed a significant trend that the proportion of tooth loss increased gradually from Q1 to Q4. The results of pairwise comparisons are also shown in Figure 2. However, there was no significant difference in the proportion of participants with tooth decay among the four groups (*χ*^2^ =6.80, *p* = 0.079).

To further verify whether the association between dental diseases and AoAC was influenced by potential confounders, we performed univariate and multivariate logistic regressions for the presence of AoAC. As shown in Table 2, tooth loss significantly showed a positive association with AoAC presence in the unadjusted model (OR: 1.289, 95% CI: 1.135–1.465, *p* < 0.001). Compared with participants without tooth loss or tooth decay, tooth loss or decay remained associated with a significantly higher prevalence of AoAC in multivariate model adjusting for demographic data, physical examination results, medical history, and biochemical risk factors (OR: 1.158, 95% CI: 1.010–1.329, *p* = 0.036). These results suggested that dental diseases were an independent risk factor for AoAC, where the presence of any dental disease increased the risk of AoAC by about 16%.

### 3.4. Dental Diseases Increase the Risk of AoAC Progression Independent of Renal Dysfunction

**Figure 3:**
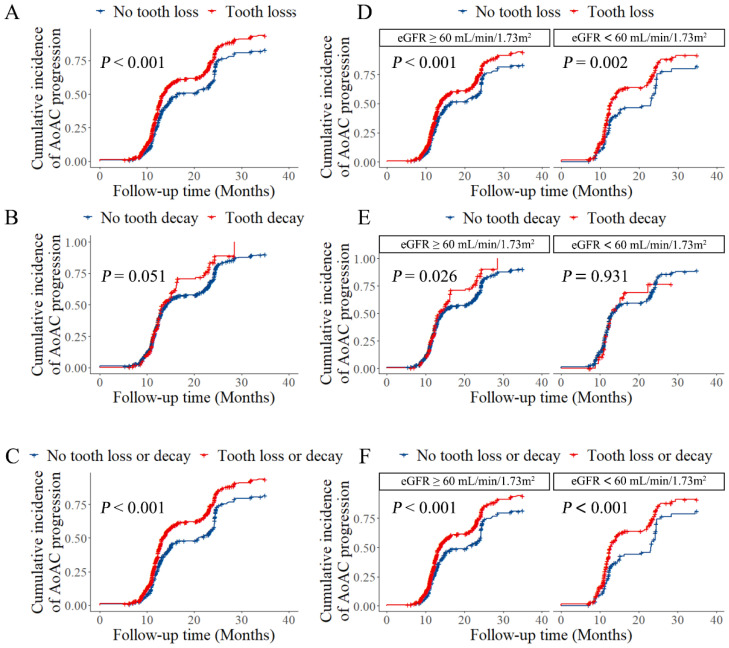
Cumulative incidences of AoAC progression by dental diseases and stratified by renal dysfunction. (**A**–**C**) show cumulative incidences of AoAC progression by tooth loss, tooth decay, tooth loss, or decay, respectively, and (**D**–**F**) show results stratified by renal dysfunction. The cumulative incidences were estimated with the Kaplan–Meier method and compared with the log-rank test.

We further analyzed the risk of AoAC progression over time by dental diseases in participants followed up during the study and with at least two X-rays. AoAC progression was defined as an increase in the AoAC score measured by X-ray. As shown in Figure 3A,C, tooth loss or decay significantly increased the risk of AoAC progression over time, and this effect may be mainly contributed by tooth loss (log-rank *p* < 0.001 for tooth loss or decay, and tooth loss alone).

In addition, the cumulative incidences of AoAC progression were estimated separately according to renal function stratification. Since AoAC is more prevalent in patients with CKD stage 3 and above, we grouped participants by eGFR using a cutoff point of 60 mL/min/1.73 m^2^. As shown in Figure 3D–F, tooth loss or decay and tooth loss alone significantly increased the risk of AoAC progression in participants from both groups. While tooth decay was observed to be associated with AoAC progression in the group with eGFR ≥ 60 mL/min/1.73 m^2^ (log-rank *p* = 0.026). Baseline characteristics of the participants included in the longitudinal analysis by the dental diseases are shown in Supplementary Tables S2–S4.

To eliminate the effect of potential confounders, we further performed multivariate Cox proportional hazard regressions for AoAC progression, stratified by important risk factors for AoAC.

**Figure 4:**
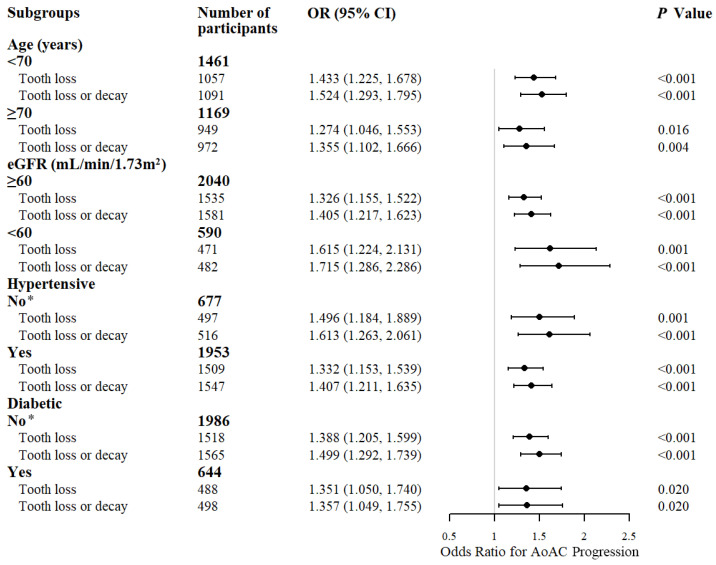
Subgroup analysis of the association of dental diseases and the progression of AoAC. Variables included in the adjusted models include age, sex, BMI, SBP, DBP, smoking, drinking, hypertension, hyperlipidemia, diabetes mellitus, TG, LDL-C, HDL-C, FPG, eGFR. OR indicates odds ratio; CI: confidence interval; BMI: body mass index; SBP: systolic blood pressure; DBP: diastolic blood pressure; TG: triglyceride; LDL-C: low-density lipoprotein cholesterol; HDL-C: high-density lipoprotein; FPG: fasting plasma glucose; eGFR: estimated glomerular filtration rate. * eGFR was removed from the adjusted variables due to violation of the proportional hazard assumption in subgroups without hypertension and diabetes mellitus. All other models met the proportional hazards assumption tested with Schoenfeld residuals.

In the fully adjusted model, and any stratification of established risk factors for AoAC, tooth loss or decay, and tooth loss alone significantly increased the risk of AoAC progression (Figure 4). These results suggested that tooth loss or decay was strong risk factors for AoAC progression, independent of recognized risk factors such as renal dysfunction (*p* < 0.001). Tooth decay was not included in the analysis due to a violation of the proportional hazard assumption.

## 4. Discussion

In this study, we aimed to investigate the association between tooth loss, tooth decay, and AoAC in a large-scale community-based population. Our findings suggested dental diseases were independent risk factors for AoAC based on both cross-section analysis and longitudinal analysis, which was not mediated by renal dysfunction. Tooth loss was a more important contributor than tooth decay. Compared with participants without tooth loss or tooth decay, any missing or decayed teeth may increase the risk of AoAC.

Tooth loss is an advanced stage of many dental diseases, including periodontitis and tooth decay, and is considered an effective marker of dental health over the life course [16,17]. Tooth loss also leads to compromised nutrition and a poorer central nervous system function [18]. Tooth loss and tooth decay have also been identified as important risk factors for cardiovascular diseases [19], and tooth loss increases the risk of major cardiovascular events such as myocardial infarction, heart failure, and stroke [20,21]. Tooth loss is in positive correlation with a positive correlation with cardiovascular mortality [22,23]. Previous research reported that tooth loss is correlated to more severe carotid and coronary artery calcification [24,25]. However, after adjusting age, sex, and other well-known risk factors for cardiovascular disease, the significant correlation was no longer present in the multivariate model. This study was based on a larger aged population in China, and identified tooth loss as an independent risk factor for AoAC progression in multivariate analyses adjusted with established AoAC risk factors.

Vascular calcification (VC) is a common and featured arterial pathological condition in chronic kidney disease and diabetes [26,27]. The dental diseases could lead to impaired kidney function [9,28], and are also associated with an increased risk of diabetes [29]. However, our results in this large community-based population suggested that tooth loss or decay was a strong risk factor for AoAC independent of established risk factors, suggesting that other underlying mechanisms remain to be further explored. 

Inflammation is an important mechanism that promotes VC [30]. Inflammatory mediators, including interleukin-1β, interleukin-6, and tumor necrosis factor (TNF)-α, could stimulate the transformation of VSMCs from a contractile phenotype to an osteogenic phenotype [31,32]. Dental diseases are associated with a significant increase in the severity of systemic inflammation [33], which may be an important mechanism linking it with VC. However, due to limitations in the range of data, we cannot provide relevant results based on systemic inflammation as evidence. On the other side, VC shares many features with bone development and metabolism. Tooth loss involves alveolar bone loss, which has been reported of a strong association with osteoporosis in previous studies and could be a local reflection of systemic bone loss [34,35]. In this study, there was a higher proportion of elderly women, who are susceptible to osteoporosis, and this proportion was even higher among participants with AoAC. Estrogen changes after menopause may be related to this phenomenon [36]. However, the study population was limited to over 60 years of age, which made it unable to further explore the influence of menopausal status.

In this study, AoAC was measured and scored based on X-rays in the large-scale population. Although CT remains the reference standard for measurement and quantification of VC, its high-cost limits applications in large-scale population examinations [37]. Since the aortic arch has been identified as the most vulnerable site for calcifications in the thoracic aorta [38], the severity of AoAC is indicated by the calcification score in the aortic node region, AoAC score was widely accepted to assess the severity of VC, which has been validated to be a simple, inexpensive, and reliable reflection of AoAC [39].

Our results may provide new perspectives for clinical practice and future research directions. Tooth loss and tooth decay are easy to observe, which may be beneficial to identifying subjects with high cardiovascular risk. Whether dental interventions could reduce VC and improve cardiovascular health needs further research. Since the mechanism of VC has not been elucidated, and there is still a lack of effective treatment, further exploration of the mechanism may provide a new perspective for the prevention and treatment of AC.

## 5. Conclusions

Dental diseases are potential predictors for AoAC in elderly people, which are independent of renal dysfunction.

## 6. Limitations

This study has several limitations. Firstly, the number of teeth lost and the severity of tooth decay was not quantified, so the dose–response relationship was not measured further. Secondly, no serological examinations reflected the level of inflammation in this study, therefore the discussion on the mechanism of inflammation lacked support from results.

## Figures and Tables

**Table 1:** Characteristics of participants according to the presence of AoAC.

	All Subjects	Non-AoAC	AoAC	*p*-Value
Number	9463	1432	8031	
**Demographics**				
Age, years	70.76 ± 5.74	69.59 ± 4.87	70.97 ± 5.86	<0.001
Female, *n* (%)	5441 (57.50)	717 (50.07)	4724 (58.82)	<0.001
SBP, mmHg	136.39 ± 17.47	134.90 ± 17.22	136.65 ± 17.50	<0.001
DBP, mmHg	77.36 ± 10.34	77.22 ± 10.39	77.39 ± 10.33	0.555
BMI, kg/m^2^	24.02 ± 3.13	24.07 ± 3.03	24.01 ± 3.15	0.497
**Medical history**				
CHD, *n* (%)	906 (9.57)	136 (9.50)	770 (9.59)	0.953
Hypertension, *n* (%)	7648 (80.82)	1082 (75.56)	6566 (81.76)	<0.001
Diabetes Mellitus, *n* (%)	1963 (20.74)	287 (20.04)	1676 (20.87)	0.499
Hyperlipidemia, *n* (%)	3627 (38.33)	470 (32.82)	3157 (39.31)	<0.001
**Dental health**				
Tooth loss, *n* (%)	7278 (76.91)	1044 (72.91)	6234 (77.62)	<0.001
Tooth decay, *n* (%)	1160 (12.26)	163 (11.38)	997 (12.41)	0.292
Tooth loss or decay, *n* (%)	7550 (79.78)	1077 (75.21)	6473 (80.60)	<0.001
**Hematology and biochemical test**				
WBC, 10^9^/L	6.20 (5.20, 7.20)	6.20 (5.39, 7.30)	6.12 (5.20, 7.20)	0.063
HGB, g/L	134.43 ± 13.51	136.04 ± 13.41	134.14 ± 13.51	<0.001
PLT, 10^9^/L	208.70 ± 50.04	209.98 ± 50.10	208.47 ± 50.03	0.294
TG, mmol/L	1.28 (0.94, 1.78)	1.29 (0.94, 1.78)	1.28 (0.94, 1.78)	0.814
TC, mmol/L	5.03 (4.34, 5.75)	4.98 (4.32, 5.67)	5.03 (4.34, 5.76)	0.075
LDL-C, mmol/L	2.90 (2.28, 3.55)	2.88 (2.32, 3.49)	2.91 (2.27, 3.56)	0.703
HDL-C, mmol/L	1.40 (1.19, 1.67)	1.38 (1.15, 1.61)	1.41 (1.20, 1.68)	<0.001
FPG, mmol/L	5.46 (4.94, 6.24)	5.42 (4.86, 6.21)	5.46 (4.96, 6.24)	0.022
eGFR, mL/min/1.73 m^2^	72.95 (64.11, 86.58)	70.21 (63.55, 77.78)	73.63 (64.36, 88.18)	<0.001
**Lifestyle**				
Smoking, *n* (%)	1526 (16.13)	243 (16.97)	1283 (15.98)	0.367
Drinking, *n* (%)	2092 (22.11)	337 (23.53)	1755 (21.85)	0.168
Physical activity hours/week, *n* (%)				0.122
<3 h	2256 (23.84)	330 (23.04)	1926 (23.98)	
3–10 h	6101 (64.47)	912 (63.69)	5189 (64.61)	
>10 h	1106 (11.69)	190 (13.27)	916 (11.41)	

Continuous variables are summarized as mean  ±  standard deviation for normally distributed, and median (25th, 75th percentiles) for non-normally distributed. Categorical variables are summarized as count (percentage). SBP indicates systolic blood pressure; DBP: diastolic blood pressure; BMI: body mass index; CHD: coronary heart disease; WBC: white blood cell count; HGB: hemoglobin; PLT: platelet; TC: total cholesterol; TG: triglyceride; LDL-C: low-density lipoprotein cholesterol; HDL-C: high-density lipoprotein cholesterol; FPG: fasting plasma glucose; eGFR: estimated glomerular filtration rate.

**Table 2:** Odds ratios for the presence of AoAC by dental diseases.

**Variables**	**OR**	**95% CI**	***p* Value**
**Unadjusted**			
Tooth loss	1.289	1.135–1.465	<0.001
Tooth decay	1.103	0.925–1.316	0.273
Tooth loss or decay	1.369	1.200–1.563	<0.001
**Model 1**			
Tooth loss	1.172	1.028–1.336	0.018
Tooth decay	1.113	0.932–1.329	0.236
Tooth loss or decay	1.248	1.090–1.428	0.001
**Model 2**			
Tooth loss	1.154	1.012–1.317	0.032
Tooth decay	1.091	0.913–1.303	0.337
Tooth loss or decay	1.223	1.068–1.401	0.004
**Model 3**			
Tooth loss	1.110	0.971–1.268	0.125
Tooth decay	1.015	0.848–1.216	0.868
Tooth loss or decay	1.158	1.010–1.329	0.036

Model 1: adjusted with age, gender, BMI, SBP, DBP, smoking, drinking. Model 2: further adjusted with hypertension, hyperlipidemia, diabetes mellitus. Model 3: further adjusted with TG, LDL-C, HDL-C.FPG, eGFR. OR indicates odds ratio; CI: confidence interval; BMI: body mass index; SBP: systolic blood pressure; DBP: diastolic blood pressure; TG: triglyceride; LDL-C: low-density lipoprotein cholesterol; HDL-C: high-density lipoprotein; FPG: fasting plasma glucose; eGFR: estimated glomerular filtration rate.

## Data Availability

Not applicable.

## Supplementary Materials

The following supporting information can be downloaded at: https://www.mdpi.com/article/10.3390/metabo12121258/s1. Figure S1 and Tables S1–S5 are available in the supplementary data.
