# Supplementary material for: Dental Diseases Increase Risk of Aortic Arch Calcification Independent of Renal Dysfunction in Older Adults: Shenzhen Community Cohort Study
Source: Metabolites. 2022 Dec 14;12(12):1258. doi: 10.3390/metabo12121258 (PMC9788133; doi:10.3390/metabo12121258)
Supplement: Supplementary file 1 [file metabolites-12-01258-s001.zip › metabolites-1985941-supplementary.pdf]

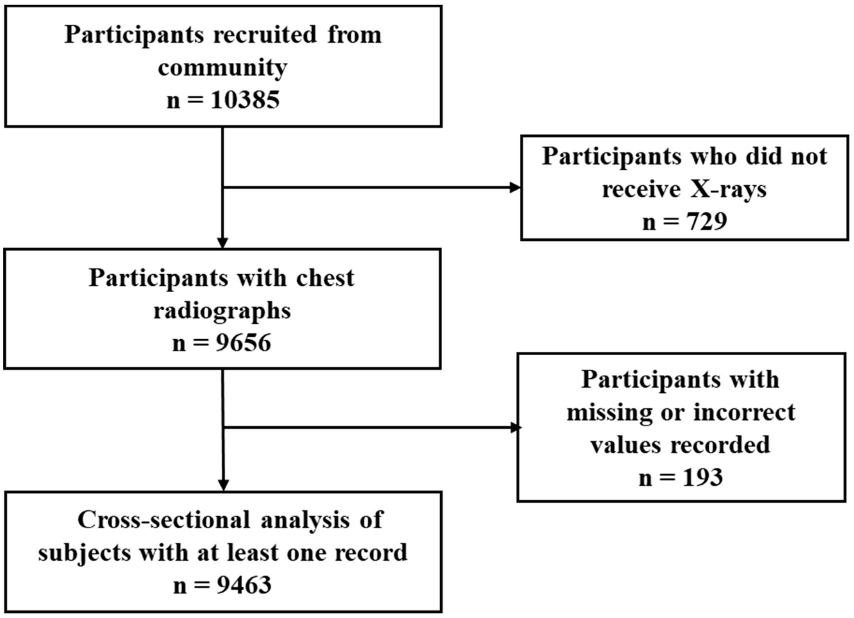

Figure S1. Flowchart of data screening and inclusion of the participants of this study.

Table S1. Effective association rules when AoAC set to consequent

Rule	Support	Confidence	Lift
Age⇒AoAC	49.04%	87.40%	1.030
Hyperlipidemia⇒AoAC	38.33%	87.04%	1.026
TC⇒AoAC	19.48%	86.82%	1.023
LDL-C⇒AoAC	26.44%	86.21%	1.016
SBP⇒AoAC	39.28%	86.15%	1.015
Tooth decay⇒AoAC	12.26%	85.95%	1.013
Hypertension ⇒AoAC	80.82%	85.85%	1.012
Tooth loss or decay ⇒AoAC	79.78%	85.74%	1.010
Tooth loss ⇒AoAC	76.91%	85.66%	1.009
DM⇒AoAC	20.74%	85.38%	1.006
TG⇒AoAC	12.37%	85.14%	1.003
FPG⇒AoAC	14.73%	85.08%	1.002

Support, Confidence and Lift of association rules are calculated based on the Apriori algorithm. "⇒" is used to indicate the direction of association rules.

SBP indicates systolic blood pressure; TC, total cholesterol; TG, triglyceride; LDL-C, low-density lipoprotein cholesterol; DM, diabetes mellitus; FPG, fasting plasma glucose.

Table S2. Baseline characteristics of participants according to presence of tooth loss

	No tooth loss	Tooth loss	*P* value
Number	624	2006	
AoAC score	0.06 (0.06, 0.12)	0.06 (0.06, 0.12)	0.602
Age, years	69.70 ± 4.95	71.26 ± 5.26	<0.001
Female, n (%)	343 (54.97)	1129 (56.28)	0.595
SBP, mmHg	135.83 ± 16.97	136.90 ± 17.58	0.183
DBP, mmHg	77.95 ± 10.03	76.83 ± 10.34	0.018
BMI, kg/m^2	24.08 ± 3.11	24.07 ± 3.17	0.929
CHD, n (%)	95 (15.22)	251 (12.51)	0.092
Hypertension, n (%)	444 (71.15)	1509 (75.22)	0.048
Diabetes Mellitus n (%)	156 (25.00)	488 (24.33)	0.773
Hyperlipidemia, n (%)	238 (38.14)	703 (35.04)	0.173
WBC, 10^9/L	6.30 (5.40, 7.40)	6.30 (5.40, 7.30)	0.88
HGB, g/L	134.86 ± 13.23	134.18 ± 13.51	0.272
PLT, 10^9/L	212.59 ± 50.73	206.76 ± 48.73	0.01
TG, mmol/L	1.33 (0.91, 1.81)	1.26 (0.93, 1.73)	0.267
TC, mmol/L	4.97 (4.35, 5.68)	5.01 (4.32, 5.74)	0.726
LDL-C, mmol/L	2.89 (2.31, 3.53)	2.93 (2.29, 3.55)	0.862
HDL-C, mmol/L	1.34 (1.15, 1.58)	1.38 (1.16, 1.63)	0.102
FPG, mmol/L	5.30 (4.75, 5.99)	5.34 (4.80, 6.11)	0.084
eGFR, ml/min/1.73m^2	67.44 (61.82, 74.02)	66.87 (60.52, 73.04)	0.031
Smoking, n (%)	82 (13.14)	301 (15.00)	0.277
Drinking, n (%)	135 (21.63)	527 (26.27)	0.023
Physical activity hours/week, n (%)			0.231
<3h	124 (19.87)	459 (22.88)	
3-10h	435 (69.71)	1328 (66.20)	
>10h	65 (10.42)	219 (10.92)	

Continuous variables are summarized as mean ± standard deviation for normally distributed, and median (25th, 75th percentiles) for non-normally distributed. Categorical variables are summarized as count (percentage).

SBP indicates systolic blood pressure; DBP, diastolic blood pressure; BMI, body mass index; CHD, coronary heart disease; WBC, white blood cell count; HGB, hemoglobin; PLT, platelet; TC, total cholesterol; TG, triglyceride; LDL-C, low-density lipoprotein cholesterol; HDL-C, high-density lipoprotein cholesterol; FPG, fasting plasma glucose; eGFR estimated glomerular filtration rate.

Table S3. Baseline characteristics of participants according to presence of tooth decay

	No tooth decay	Tooth decay	*P* value
Number	2371	259	
AoAC score	0.06 (0.06, 0.12)	0.12 (0.06, 0.12)	0.551
Age, years	70.89 ± 5.28	70.86 ± 4.79	0.912
Female, n (%)	1323 (55.80)	149 (57.53)	0.641
SBP, mmHg	136.43 ± 17.36	138.66 ± 18.09	0.05
DBP, mmHg	76.96 ± 10.16	78.33 ± 11.26	0.042
BMI, kg/m^2	24.08 ± 3.14	23.97 ± 3.30	0.589
CHD, n (%)	317 (13.37)	29 (11.20)	0.376
Hypertension, n (%)	1765 (74.44)	188 (72.59)	0.566
Diabetes Mellitus n (%)	589 (24.84)	55 (21.24)	0.228
Hyperlipidemia, n (%)	854 (36.02)	87 (33.59)	0.48
WBC, 10^9/L	6.30 (5.40, 7.40)	6.20 (5.30, 7.20)	0.439
HGB, g/L	134.30 ± 13.55	134.71 ± 12.45	0.641
PLT, 10^9/L	207.83 ± 49.36	211.05 ± 48.30	0.318
TG, mmol/L	1.28 (0.93, 1.78)	1.21 (0.92, 1.62)	0.177
TC, mmol/L	4.99 (4.32, 5.74)	5.05 (4.34, 5.70)	0.58
LDL-C, mmol/L	2.93 (2.29, 3.54)	2.88 (2.36, 3.63)	0.433
HDL-C, mmol/L	1.37 (1.16, 1.61)	1.41 (1.19, 1.65)	0.226
FPG, mmol/L	5.33 (4.79, 6.09)	5.29 (4.78, 6.01)	0.522
eGFR, ml/min/1.73m^2	66.95 (60.70, 73.23)	67.20 (62.00, 73.33)	0.43
Smoking, n (%)	338 (14.26)	45 (17.37)	0.208
Drinking, n (%)	578 (24.38)	84 (32.43)	0.006
Physical activity hours/week, n (%)			0.946
<3h	527 (22.23)	56 (21.62)	
3-10h	1587 (66.93)	176 (67.95)	
>10h	257 (10.84)	27 (10.42)	

Continuous variables are summarized as mean ± standard deviation for normally distributed, and median (25th, 75th percentiles) for non-normally distributed. Categorical variables are summarized as count (percentage).

SBP indicates systolic blood pressure; DBP, diastolic blood pressure; BMI, body mass index; CHD, coronary heart disease; WBC, white blood cell count; HGB, hemoglobin; PLT, platelet; TC, total cholesterol; TG, triglyceride; LDL-C, low-density lipoprotein cholesterol; HDL-C, high-density lipoprotein cholesterol; FPG, fasting plasma glucose; eGFR estimated glomerular filtration rate.

Table S4. Baseline characteristics of participants according to presence of tooth loss or decay

	No tooth loss or decay	Tooth loss or decay	*P* value
Number	567	2063	
AoAC score	0.06 (0.06, 0.12)	0.06 (0.06, 0.12)	0.826
Age, years	69.66 ± 5.05	71.23 ± 5.23	<0.001
Female, n (%)	313 (55.20)	1159 (56.18)	0.713
SBP, mmHg	135.94 ± 16.94	136.84 ± 17.57	0.273
DBP, mmHg	77.93 ± 10.04	76.87 ± 10.33	0.03
BMI, kg/m^2	24.14 ± 3.11	24.05 ± 3.16	0.584
CHD, n (%)	91 (16.05)	255 (12.36)	0.026
Hypertension, n (%)	406 (71.60)	1547 (74.99)	0.115
Diabetes Mellitus n (%)	146 (25.75)	498 (24.14)	0.463
Hyperlipidemia, n (%)	218 (38.45)	723 (35.05)	0.148
WBC, 10^9/L	6.30 (5.40, 7.40)	6.30 (5.40, 7.30)	0.912
HGB, g/L	134.90 ± 13.30	134.19 ± 13.49	0.264
PLT, 10^9/L	212.84 ± 51.30	206.86 ± 48.62	0.01
TG, mmol/L	1.33 (0.91, 1.83)	1.26 (0.93, 1.73)	0.322
TC, mmol/L	4.98 (4.39, 5.71)	5.00 (4.30, 5.74)	0.681
LDL-C, mmol/L	2.94 (2.36, 3.54)	2.92 (2.29, 3.55)	0.648
HDL-C, mmol/L	1.35 (1.15, 1.58)	1.38 (1.16, 1.63)	0.208
FPG, mmol/L	5.32 (4.76, 6.02)	5.33 (4.79, 6.10)	0.34
eGFR, ml/min/1.73m^2	67.66 (61.82, 74.02)	66.81 (60.53, 73.06)	0.038
Smoking, n (%)	69 (12.17)	314 (15.22)	0.079
Drinking, n (%)	121 (21.34)	541 (26.22)	0.02
Physical activity hours/week, n (%)			0.097
<3h	109 (19.22)	474 (22.98)	
3-10h	401 (70.72)	1362 (66.02)	
>10h	57 (10.05)	227 (11.00)	

Continuous variables are summarized as mean ± standard deviation for normally distributed, and median (25th, 75th percentiles) for non-normally distributed. Categorical variables are summarized as count (percentage).

SBP indicates systolic blood pressure; DBP, diastolic blood pressure; BMI, body mass index; CHD, coronary heart disease; WBC, white blood cell count; HGB, hemoglobin; PLT, platelet; TC, total cholesterol; TG, triglyceride; LDL-C, low-density lipoprotein cholesterol; HDL-C, high-density lipoprotein cholesterol; FPG, fasting plasma glucose; eGFR estimated glomerular filtration rate.

Table S5. Baseline characteristics of participants according to presence of tooth loss or decay

Variables	HR	95% CI	*P* value
**Model 1**			
Tooth loss	1.377	1.221-1.553	<0.001
Tooth loss or decay	1.458	1.286-1.652	<0.001
**Model 2**			
Tooth loss	1.352	1.199-1.525	<0.001
Tooth loss or decay	1.433	1.265-1.625	<0.001
**Model 3**			
Tooth loss	1.356	1.202-1.530	<0.001
Tooth loss or decay	1.437	1.267-1.629	<0.001

Model 1: adjusted with age, gender, BMI, SBP, DBP, smoking, drinking.

Model 2: further adjusted with hypertension, hyperlipidemia, diabetes mellitus

Model 3: further adjusted with TG, LDL-C, HDL-C.FPG, eGFR.

HR indicates hazard ratio; CI, confidence interval; BMI, body mass index; SBP, systolic blood pressure; DBP, diastolic blood pressure; TG, triglyceride; LDL-C, low-density lipoprotein cholesterol; HDL-C, high-density lipoprotein; FPG, fasting plasma glucose; eGFR, estimated glomerular filtration rate.
